# Having Multiple Sexual Partners among Iranian Intra-Venous Drug Users

**DOI:** 10.3389/fpsyt.2014.00125

**Published:** 2014-10-10

**Authors:** Shervin Assari, Mosaieb Yarmohamadivasel, Maryam Moghani Lankarani, Mahmood Sehat, Hooman Narenjiha, Hassan Rafiey, Roya Noori, Peymaneh Shirinbayan, Khodabakhsh Ahmadi

**Affiliations:** ^1^Department of Health Behavior and Health Education, University of Michigan School of Public Health, Ann Arbor, MI, USA; ^2^Center for Research on Ethnicity, Culture, and Health, University of Michigan School of Public Health, Ann Arbor, MI, USA; ^3^Department of Psychiatry, University of Michigan School of Medicine, Ann Arbor, MI, USA; ^4^Social Determinant of Health Research Center, University of Social Welfare and Rehabilitation Sciences, Tehran, Iran; ^5^Department of Psychology, Bu-Ali Sina University, Hamedan, Iran; ^6^Universal Network for Health Information Dissemination and Exchange, Tehran, Iran; ^7^Medicine and Health Promotion Institute, Tehran, Iran; ^8^Substance Abuse and Dependence Research Center, University of Social Welfare and Rehabilitation Sciences, Tehran, Iran; ^9^Center for Behavioral and Social Research, Darius Institute, Tehran, Iran; ^10^Pediatric NeuroRehabilitation Research Center, University of Social Welfare and Rehabilitation Sciences, Tehran, Iran; ^11^Behavioral Sciences Research Center, Baqiyatallah Medical Sciences University, Tehran, Iran

**Keywords:** high-risk behaviors, sexual behaviors, Iran, injection drug users, HIV infection, multiple sexual partnership

## Abstract

**Background:** Transmission of HIV from intra-venous drug users (IDUs) to the community occurs predominantly through high-risk sexual behaviors. Limited information exists regarding the high-risk sexual behaviors of IDUs in Iran.

**Aim:** The aim of this study was to determine the prevalence and factors associated with having multiple sexual partners among Iranian IDUs.

**Methods:** This is a national survey on drug-dependent adults. Participants were sampled from medical centers, prisons, and streets of capitals of 29 provinces in Iran between May 2007 and February 2008. We analyzed data of 1416 current IDUs. Socio-demographics and drug use characteristics were entered into a binary logistic regression model to determine predictors of having multiple sexual partners.

**Results:** Having multiple sexual partners in the past or at the time of survey was reported by 56.4% of Iranian IDUs. Multivariate analysis showed that the likelihood of having multiple sexual partners in IDUs decreased by being married [odds ratio (OR), 0.38; *P* < 0.001] and increased by female gender (OR, 13.44; *P* = 0.02), having illegal income (OR, 1.72; *P* = 0.003), higher monthly family income (OR, 1.01; *P* = 0.003), pleasure, curiosity, and recreation as cause of first drug use (OR, 1.37; *P* = 0.04), ruins as usual place for injection (OR, 1.89; *P* = 0.001) and history of syringe sharing (OR, 1.50; *P* = 0.02).

**Conclusion:** Having multiple sexual partners was reported by majority of Iranian IDUs, and this was linked to socio-demographics, initiation data, and other risk behaviors. This information should be considered in prevention efforts to reduce sexual transmission of HIV infection in Iran.

## Introduction

Iran, a country of over 70 million people, lies on the key drug trafficking routes and has the highest per capita opium consumption globally (1). The national government has estimated 1.1 million severely dependent opium users nationwide (2). Of these, 200,000 are believed to be intra-venous drug users (IDUs) (3).

Prevalence of HIV among Iranian IDUs is up to 4.2%, a rate about 20,000 times higher than the prevalence among general population (4). According to the official report of the Center for Disease Control, Ministry of Health and Medical Education, in 2006, injection drug use is the main route for transmission of HIV in Iran (4). High-risk sexual behaviors are also a major contributor of HIV transmission in the country.

The high-risk sexual behavior of IDUs is an important public health risk of spreading HIV in the communities (5). There is some evidence suggesting that this risk may be an even greater risk than high-risk injecting behaviors (6). While high-risk injecting behaviors pose other IDUs and “co-injectors” to risk of HIV infection, unprotected sex poses injectors, and non-injectors to this infection. High-risk sexual behaviors of IDUs may be more prevalent than general population, especially by means of multiple sexual partners (7, 8) transmission of HIV can become out of control.

In different countries, different socio-demographic factors may be associated with risk-taking behaviors among IDUs (9–11). Unfortunately, in Iran, only a few studies have focused on sexual risk behaviors of IDUs, thus our knowledge is limited in this regard (3, 12, 13). Our limited knowledge regarding sexual high-risk behaviors of Iranian IDUs is partly due to the fact that sex is taboo in Iran, and sex outside the context of marriage is criminalized (14).

The aim of the current study was to determine the prevalence and factors associated with having multiple sexual partners among Iranian IDUs.

## Participants and Methods

### Design settings

This is a cross-sectional survey on individuals as the Rapid Situation Assessment, performed by the Darius Institute, University of Social Welfare and Rehabilitation Sciences, Tehran, Iran, in 29 provinces of Iran, in 2007.

### Codes of ethics

The study was approved by the ethical review committee of the University of Social Welfare and Rehabilitation Sciences. Informed consent was obtained from all the participants after they had been verbally reassured that the information would be kept confidential, especially from the correctional system. This study was conducted under the financial aid of the Drugs Control Headquarters. Some other reports have been extracted from this database (15–17).

### Participants and sampling

The participants were substance-dependent individuals, according to the Diagnostic and Statistical Manual of Mental Disorders, fourth Edition, and sampled from drug abuse treatment centers, prisons, and streets of the capitals of 29 provinces in Iran. The samples from drug abuse treatment centers (free of charge and paid) were selected randomly from among newly admitted individuals. Prisons sampling was also carried out randomly from among those who were registered into the prison within the previous 30 days. The snowball approach was used to take samples from streets by asking each participant to introduce at least one other participant with drug dependency. The number of samples taken from every province was proportional to the whole population of the province. The sampling was started in April 2007 and lasted for 5 months. This sampling method has been used as the main sampling strategy of drug use recommended by the Drugs Control Headquarters.

### Interview

All interviews were carried out by university graduates with bachelor or master degrees, who were dispatched to the provinces after being trained through workshops in Tehran. Each interview took between 60 and 90 min. Data were collected using a paper-based questionnaire, namely Inventory for Drug Dependency-IV, which was the modified version of the one used in the previous national Rapid Situation Assessment of Iran (18). The revision was done through a series of expert panel meetings, and new items and questions were added that met the desired objectives. Data included in this study were the following: (1) socio-demographic data: age, gender, age at the beginning of dependency, age at the beginning of injection, duration of injection, educational level, marital status, living place, status of accommodation, status of employment, living alone or with others, income (legal and illegal), drug sell income, monthly family income, and family history of drug use; (2) drug-related data: monthly expenses for buying the dominant substance, first place of drug use, first situation of drug use, main reason for first drug use, and history of drug problems treatment; and (3) injection-related data: first place of injection, situation of first injection, cause of first injection, frequency of injection in the past years, usual place of injection, and injection alone or with others; and (4) non-sexual high-risk behaviors: history of arrest, history of imprisonment, and syringe sharing.

### Outcome

The main outcome in this study was having multiple sexual partners, which was defined as having more than one sexual partner sometime in the past or at the time of the study. Most studies in this field have measured multiple sexual partners in the previous month or year. The same outcome has also been used with different time periods in Asia, the United States, and Europe. This outcome has been shown to be associated with increased risk of HIV infection (19–22).

### Statistical analysis

Data were analyzed using the SPSS software (Statistical Package for the Social Sciences, version 13.0, SPSS Inc., Chicago, IL, USA). In order to present continuous data, median [percentile 25% (Q1) and percentile 75% (Q3)] or mean ± SD were used. In order to compare categorized variables between IDUs with and without multiple sexual partners, chi-square test was used. Comparison of age between groups was done with the independent samples *t* test. Monthly family income and expenditures of drug use were compared between the two groups using the Mann–Whitney test. Multivariate stepwise logistic regression was used to determine the predictors of having multiple sexual partners. A *P* value <0.05 was considered significant.

## Results

### Participants

The mean age of the participants at the time of the study, first drug use, and first injection were 31.3 ± 8.3, 18.6 ± 5.4, and 25.9 ± 6.7 years, respectively. Of all participants, 1362 (96.5%) were men, 1388 (99.5%) were Muslims, and 1246 (93.2%) lived in urban areas. There were 783 (55.7%) single, 406 (28.9%) married, and 217 (15.4%) separated, divorced, or widowed participants. One hundred one participants (7.3%) had academic educational degrees. Of 1416 currently IDUs, 796 (56.4%) reported to have the experience of multiple sexual partners.

### Associates of having multiple sexual partners

#### Socio-demographic variables

The mean age at first drug use was lower in those who reported multiple sexual partners compared to those who did not (18.1 ± 5.5 versus 19.2 ± 5.2 years, *P* < 0.001), and the duration of injection was longer (8.0 ± 6.4 versus 6.6 ± 6.1 years, *P* < 0.001). The IDUs who reported multiple sexual partners reported a higher median monthly family income [916 ppp$ (Q1, 440 ppp$; Q3, 1466 ppp$) versus 733 ppp$ (Q1, 366 ppp$; Q3, 1210 ppp$), *P* < 0.001] and a larger amount of money spent on drugs [623 ppp$ (Q1, 366 ppp$; Q3, 1100 ppp$) versus 550 ppp$ (Q1, 330 ppp$; Q3, 990 ppp$), *P* = 0.002] than those who did not. The association between having multiple sexual partners and socio-demographic data are presented in Table 1.

**Table 1:** **The comparison of lifetime extramarital sexual relation and past year extramarital sexual relation between socio-demographic and drug-related variables**.

		Lifetime having multiple sexual partners (*N*)	
		Number	Sig.
Gender	Male	768 (56.4%)	0.917
	Female	28 (57.1%)	
Religious type	Muslim	777 (56.1%)	0.480
	Other	3 (42.9%)	
Living place	Urban	692 (55.8%)	0.036
	Rural	61 (67.0%)	
Education level	Illiterate or were barely able to read and write	69 (50.4%)	0.228
	Primary school to diploma	653 (57.2%)	
	Upper diploma	53 (52.5%)	
Marital status	Single	480 (61.6%)	0.002
	Married	163 (40.1%)	
	Separate, divorce, and widow	149 (69.0%)	
Homeless	No	637 (54.4%)	<0.001
	Yes	121 (66.5%)	
Alone living	No	628 (54.2%)	<0.001
	Yes	168 (66.7%)	
Jobless	No	431 (52.3%)	<0.001
	Yes	365 (62.2%)	
Drug income	No	508 (51.2%)	<0.001
	Yes	273 (71.1%)	
Job income	No	398 (61.0%)	0.002
	Yes	383 (52.8%)	
Illegal income	No	462 (49.8%)	<0.001
	Yes	319 (71.0%)	
Substance use by parents	No	550 (53.3%)	<0.001
	Yes	246 (64.9%)	
Substance use by members of family	No	399 (49.9%)	<0.001
	Yes	397 (65.0%)	
Beginning drug use for pleasure/enjoyment/recreation/curiosity	Yes	486 (59.6%)	0.009
	No	311 (53.3%)	
Usually inject alone	Yes	576 (56.5%)	0.571
	No	229 (57.8%)	
Injection one per day or higher	No	77 (51.6%)	0.22
	Yes	661 (56.8%)	
Usual injection in own home	No	372 (56.7%)	0.967
	Yes	433 (57.0%)	
Usual injection in friend’s home	No	546 (55.8%)	0.148
	Yes	259 (59.1%)	
Usual injection in park	No	667 (56.2%)	0.255
	Yes	138 (60.3%)	
Usual injection in school	No	800 (56.9%)	0.958
	Yes	5 (55.6%)	
Usual injection in street and lane	No	638 (55.0%)	0.004
	Yes	167 (65.0%)	
Usual injection in ruins	No	530 (52.9%)	<0.001
	Yes	275 (66.4%)	
Usual injection in soldiers’ camp	No	798 (56.7%)	0.195
	Yes	7 (77.8%)	
Usual injection in student house	No	799 (56.9%)	0.900
	Yes	6 (54.5%)	
Usual injection in work place	No	717 (56.7%)	0.625
	Yes	88 (58.3%)	
Usual injection in prison	No	747 (55.5%)	<0.001
	Yes	58 (84.1%)	

#### Drug use and injection-related variables

Participants with multiple sexual partners were more likely to be multiple drug users than those without multiple sexual partners (61.8 versus 54.4%, *P* = 0.04), but history of treatment of drug use problems was not more frequent in this group (56.4 versus 56.5%, *P* = 0.98). The associations between having multiple sexual partners with socio-demographic and drug use data are presented in Table 1.

#### High-risk behaviors

Participants with multiple sexual partners were more likely to have a history of non-fatal overdose (61.4 versus 50.0%, *P* < 0.001), non-fatal overdose in the past year (62.6 versus 54.1%, *P* = 0.005), arrest in the past year (60.0 versus 51.1%, *P* = 0.001), imprisonment in the past year (62.5 versus 49.9%, *P* < 0.001), and syringe sharing (66.7 versus 51.6%, *P* < 0.001) than those without multiple sexual partners.

### Logistic regression

Multivariate analysis showed that the likelihood of the lifetime having multiple sexual partners in IDUs increased by female gender [odds ratio (OR), 13.44; 95% confidence interval (CI), 1.30–38.90; *P* = 0.02], reporting to have illegal income (OR, 1.72; 95% CI, 1.20–2.48; *P* = 0.003), higher monthly family income (OR, 1.001; 95% CI, 1.000–1.001; *P* = 0.003), pleasure, curiosity, and recreation as cause of the first drug use (OR, 1.37; 95% CI, 1.07–1.94; *P* = 0.04), ruins as usual place of injection (OR, 1.89; 95% CI, 1.29–2.76; *P* = 0.001), and history of syringe sharing (OR, 1.500; 95% CI, 1.04–2.15, *P* = 0.02). The likelihood of having a lifetime of multiple sexual partners decreased by being married (OR, 0.38; 95% CI, 0.26–0.56; *P* < 0.001; Table 2).

**Table 2:** **Logistic regression of lifetime having multiple sexual partners**.

	Sig.	OR	95% Confidence interval for odds	
			Lower	Upper
Gender (female)	0.029	13.446	1.302	38.90
Married/single	<0.001	0.384	0.261	0.565
Illegal income	0.003	1.729	1.202	2.488
Family income	0.003	1.001	1.000	1.001
Cause of first drug use (pleasure/enjoyment, curiosity, recreation)	0.049	1.376	1076	1.941
Place of usual injection (ruins/other place)	<0.001	1.897	1.299	2.769
Syringe sharing	0.026	1.504	1.049	2.155

## Discussion

According to this study, more than 56% of Iranian IDUs report having multiple sexual partners in their lifetime. Multivariate analysis showed that the likelihood of multiple sexual partners in IDUs increased by female gender (OR, 13.44), having illegal income (OR, 1.72), higher monthly family income (OR, 1.001), pleasure/enjoyment, curiosity, and recreation as the cause of first drug use (OR, 1.37), ruins as the usual place of injection (OR, 1.89), and history of syringe sharing (OR, 1.500), while being married decreased the likelihood of having this behavior (OR, 0.38).

Recently, some studies have suggested a shift in the main mode of HIV/AIDS transmission in the country from unsafe injecting behavior to unsafe sexual relationship (3, 23–27).

In Iran, few studies have focused on the sexual risk behaviors of IDUs. Ahmadi et al., in 2012 showed that 19% of female sex workers reported at least one occasion of unprotected sex with IDU(s) in the month preceding the study. The study suggested that age, marital status, living condition, HIV knowledge, and perceived behavioral control did not affect the odds of FSWs having sex with IDUs; however, perceived HIV risk could be a target for harm reduction interventions amongst Iranian female sex workers who may be at risk of HIV due to sex with IDUs in Iran (28). In another study of 360 male heterosexual IDUs who were sampled from streets of eight different geographical parts of Iran, Mirabi and coworkers showed that about 21% of male IDUs reported unprotected anal intercourse during the past month. Although HIV knowledge was not linked to unprotected anal intercourse, age, marital status, and perceived HIV risk were associated with the likelihood of unprotected anal intercourse (29). Assari and coworkers in a recent study analyzed data of 1131 male sexually active IDUs. They showed that 83.3% of sexually active IDUs reported inconsistent condom use. In that study, likelihood of inconsistent condom use was higher among those with a history of syringe sharing, but lower among those with higher education levels, those who mostly inject at home, and those with a history of treatment. Authors emphasized the need for combined programs targeting both sexual and injection behavior among Iranian IDUs (30). Rafiey et al. also reported a positive association between high-risk injecting and high-risk sexual behaviors among Iranian IDUs (16).

In one study from Vietnam in 1999, having multiple sexual partners within the past 6 months was reported by 44% of single and 24% of married IDUs (31). A Chinese study showed that 25% of IDUs had multiple partners and 48% had IDU partners (7). Another study from Indonesia reported that 48% of IDUs had multiple sexual partners, and 40% of male IDUs had sexual relations with a female sex worker in the past 12 months (32).

A link between female gender and having multiple sexual partners has been reported, which can be explained by the fact that female IDUs can use sex to obtain money in order to feed their drug habits (33–39). In concordance with our study, Williams and colleagues showed that women were more likely to report a higher number of sexual partners (40). However, contradictory results have also been published (6). One domestic qualitative study on IDUs in Iran showed that women compared to men reported higher rates of illegal sexual relations and sex for money or drugs (41). According to these results, the risk of HIV infection transmission might be higher through sexual contact of female IDUs. There are a number of reasons why female IDUs may engage in more risky behaviors. Women may be more stigmatized for the drug use, and stigmatization may lead to more high-risk behaviors (42). Another explanation is female IDUs in each country may act as prostitutes who have resorted to sex in order to pay for drugs.

Having multiple sexual partners may be higher in single than married drug users (31). Our study also showed that marriage was a protective factor that lowered the likelihood of having multiple sexual partners. We also demonstrated that having multiple sexual partners was linked to higher family income and income from illegal sources. We found no supporting evidence in the literature; however, there are reports regarding an association between illegal income and other high-risk behaviors such as drug use, drug injection, and syringe sharing (43–45). The link between family income and having multiple sexual partners was reported alcohol users (46), but there is no report on IDUs. Interestingly, according to population-based surveys in industrialized countries, men of low socioeconomic status reported fewer sexual partners than men of high socioeconomic status (47). In another study, factors associated with higher seroprevalence of HIV-1 included relatively high socioeconomic status (48).

We found that having multiple sexual partners was linked to syringe sharing. In line with our study, there are studies showing an association between high-risk sexual behaviors and sharing of injection equipment (49–51). We found that IDUs whose usual place of injection was ruins had a higher chance of having multiple sexual partners. We found no information in the literature in this regard, although it is known that injection-related risk behavior may be higher in public place injectors (52).

Having multiple sexual partners increases the chance of sexually transmitted infections such as HIV. The Rapid Situation Assessment 1998/1999 showed that 20% of respondents had not even heard of HIV/AIDS. Between 20 and 30% of those who had heard of the disease were unaware that it could be transmitted by sharing injecting equipment (18). At that time, there had been little work on HIV/AIDS prevention for drug injectors. In 1999, it was reported that there were no printed materials on HIV/AIDS available to drug users at all (53). Harm reduction in IDUs should not only minimize the high-risk injecting behavior but also high-risk sexual behavior. However, currently in Iran, most of the attention of harm reduction for IDUs is spent on safer injection. In other countries also, control of sexual behavior of IDUs seems to be harder than control of their high-risk sexual behavior (50).

Educational programs should target sexual behaviors of IDUs. One of the findings of this study, for example, was that female IDUs may need extra attention for interventions to improve their sexual behavior. The information provided here can be used by program planners, policy makers in the field of HIV/AIDS, or drug use to design sexual risk minimization programs and reduce HIV transmission via IDUs. Our finding also determined the need for further prospective studies, which will decrease the chance of high-risk sexual behavior, focusing on predictors of having multiple sexual partners proposed here. Unfortunately, very few studies have focused on high-risk behaviors of IDUs in Iran (28, 29, 54–57).

There were some limitations to this study. First, we relied on participants’ self-reported data, which accompany with recall bias. Second, sexual behavior is a major taboo in Iran, and individuals who participated in the study may under-report having multiple sexual partners. Third, because of the cross-sectional design of this study, it is not possible to draw a conclusion on the direction of the associations. Fourth, this study did not measure other aspects of sexual behavior such as same-sex sexual behaviors or unprotected sex. Lastly, the number of women in our study was too low to draw a strong conclusion. This study operationalized multiple sexual partners as a binary outcome. Number of lifetime partners in a specific time period may be a more important risk factor for HIV risk, however, was not considered in this study.

## Conclusion

High prevalence of having multiple sexual partners among Iranian IDUs is a threat to public health in Iran. High-risk sexual behaviors of IDUs contribute to the spread of HIV infection from a concentrated epidemic to a non-concentrated epidemic in the society. Socio-demographic and injection-related characteristics are linked to sexual risk behaviors of IDUs. This information can be used in prevention efforts to control HIV/AIDS infection and other sexually transmitted infections in Iran.

## Conflict of Interest Statement

The authors declare that the research was conducted in the absence of any commercial or financial relationships that could be construed as a potential conflict of interest.
